# Estimating misclassification error: a closer look at cross-validation based methods

**DOI:** 10.1186/1756-0500-5-656

**Published:** 2012-11-28

**Authors:** Songthip Ounpraseuth, Shelly Y Lensing, Horace J Spencer, Ralph L Kodell

**Affiliations:** 1 Department of Biostatistics, University of Arkansas for Medical Sciences, 4301 W. Markham St. Slot 781, Little Rock, AR, 72205, USA

**Keywords:** Cross-validation, Bootstrap Cross-validation, Classification Error Estimation, Mean Squared Error

## Abstract

**Background:**

To estimate a classifier’s error in predicting future observations, bootstrap methods have been proposed as reduced-variation alternatives to traditional cross-validation (CV) methods based on sampling without replacement. Monte Carlo (MC) simulation studies aimed at estimating the true misclassification error conditional on the training set are commonly used to compare CV methods. We conducted an MC simulation study to compare a new method of bootstrap CV (BCV) to *k*-fold CV for estimating clasification error.

**Findings:**

For the low-dimensional conditions simulated, the modest positive bias of *k*-fold CV contrasted sharply with the substantial negative bias of the new BCV method. This behavior was corroborated using a real-world dataset of prognostic gene-expression profiles in breast cancer patients. Our simulation results demonstrate some extreme characteristics of variance and bias that can occur due to a fault in the design of CV exercises aimed at estimating the true conditional error of a classifier, and that appear not to have been fully appreciated in previous studies. Although CV is a sound practice for estimating a classifier’s *generalization error*, using CV to estimate the *fixed misclassification error* of a trained classifier conditional on the training set is problematic. While MC simulation of this estimation exercise can correctly represent the average *bias* of a classifier, it will overstate the between-run *variance* of the bias.

**Conclusions:**

We recommend *k*-fold CV over the new BCV method for estimating a classifier’s generalization error. The extreme negative bias of BCV is too high a price to pay for its reduced variance.

## Findings

### Background

Class prediction involves the use of statistical learning techniques to develop algorithms for classifying unknown samples through supervised learning on samples of known class. In assessing the performance of a classification algorithm, the goal is to estimate its ability to generalize, i.e., to predict the outcomes of samples not included in the data set used to train the classifier. The performance may be assessed on the basis of a number of different indices. For problems having a dichotomous outcome variable (e.g., positive or negative), the sensitivity, specificity, positive predictive value and negative predictive value are indices that may be of interest in addition to the overall prediction accuracy [1]. In this paper, attention is focused on the overall prediction accuracy, or equivalently, on its counterpart, the prediction error.

Cross-validation (CV) is a widely used method for performance assessment in class prediction [2-4]. With *k*-fold CV, a data set of *n* samples is randomly divided into *k* subsets each having (approximately) *n*/*k* samples. Each of these *k* subsets serves in turn as a test set. For each of these *k* test sets of size *n*/*k*, a classifier is trained on the remaining (*k*-1)×(*n*/*k*) observations (the training set). The trained classifier is then used to classify the *n*/*k* samples in the test set, and the prediction error (perhaps, along with other indices) is calculated. The combined value of the prediction error over the *k* test sets, which is based on the prediction of all *n* samples one time each, is the cross-validated estimate of that error. Generally, several replicates of *k*-fold cross-validation are performed based on different random permutations of the *n* samples in order to account for the random resampling variance, and the average and standard deviation of these replicates are used to assess the performance of the classifier [5,6]. When *k* = *n*, the exercise is called leave-one-out cross-validation (LOOCV); there is only one unique way to do LOOCV and, hence, it cannot be replicated. A common choice of *k* is 10, and 10 to 30 replicates of 10-fold CV have been shown to be sufficient to achieve stable values of the prediction error [7].

Before 10-fold CV became popular, efforts were directed toward reducing the variability of LOOCV, recognizing that it gave nearly unbiased estimates of the prediction error [8]. The .632 and .632+ bootstrap methods are well known alternatives to LOOCV [9]. Recently, Fu, Carroll and Wang [10] introduced a new bootstrap version of LOOCV (bootstrap cross-validation or BCV), which they compared to LOOCV and to the .632 bootstrap method (BT632) on problems with low-dimensional predictor spaces. Like Efron and Tibshirani [9], Fu et al. [10] used a mean squared error (MSE) represented by the mean squared bias (MSB) over *N* Monte Carlo simulations (discussed in Methods Section) as the primary criterion for evaluating estimators of the *true conditional error*, i.e., the true misclassification error of the trained classifier conditional on the training set [9]. These and similar investigations into estimating the true conditional error via cross-validation (e.g., see [7,11]) have been interpreted as assessing a classifier’s error in predicting future observations, i.e., its generalization error [8,9]. It is argued in this paper that while cross-validation is a sound, generally-accepted method for evaluating a classifier’s generalization error, it may be problematic to use cross-validation to assess this generalizability in terms of estimating a true conditional error defined as a single fixed quantity for a given set of data. With that approach the variance of cross-validation will tend to be overstated, even though its bias can still be appropriately characterized, as will be shown in this paper via Monte Carlo simulation and will be explained more fully in the Discussion.

While Efron and Tibshirani used the traditional absolute scale to calculate the MSB and its square root (the root mean square or RMS in their notation), Fu et al. focused on what they termed the mean squared ‘relative’ error (MSRE), stating that calculations on the absolute scale gave similar results. Here, the mean squared error and associated quantities calculated on the absolute scale are used.

The purpose of this paper is to report the results of a more extensive comparison of BCV to conventional CV done via a simulation study like that of Fu et al. [10], based on *k*-fold CV in addition to LOOCV, and to use those results to fuel a discussion of several issues related to cross-validation. Finally, the performance of BCV and *k*-fold CV are demonstrated for a real-world data set by classifying patients with breast cancer according to prognosis based on their gene-expression profiles.

### Methods

#### Mean squared error

In order to facilitate the definition of terms, suppose for the moment that *k*-fold cross-validation (*k*CV) will be used to assess a classifier’s true conditional error rate based on the results of *N* Monte Carlo simulations. Let *k* <*n*, where *n* is the sample size, and assume that *k*CV is repeated *R* times. Then the MSE for the *i*^th^ simulation is given by

(1) $$\begin{array}{l} MSE=\frac{1}{R}\sum_{r=1}^{R}{\left({ê}_{\mathrm{ri}}-e_{i}\right)}^{2}\\ \;=\frac{1}{R}\sum_{r=1}^{R}{\left({ê}_{\mathrm{ri}}-{\overset{―}{e}}_{\mathrm{Ri}}\right)}^{2}+{\left({\overset{―}{e}}_{\mathrm{Ri}}-e_{i}\right)}^{2}\text{,} \end{array}$$

where *e*_*i*_ denotes the true conditional error for the *i*^th^ simulation, ${ê}_{\mathrm{ri}}$ denotes the *r*^th^*k*CV estimate of *e*_*i*_ for the *i*^th^ simulation, and ${\overset{―}{e}}_{\mathrm{Ri}}=\left(1/R\right)\sum_{r=1}^{R}{ê}_{\mathrm{ri}}$ is the mean estimate of the *i*^th^ true conditional error over *R* re-samples. The terms on the right hand side of (1) are the variance and bias components of the MSE. The average MSE over *N* simulations is given by

(2) $$\overset{―}{MSE}=\frac{1}{N}\sum_{i=1}^{N}\frac{1}{R}\sum_{r=1}^{R}{\left({ê}_{\mathrm{ri}}-e_{i}\right)}^{2}\text{,}$$

which can be decomposed into average variance and average (squared) bias components,

(3) $$\begin{array}{l} \overset{―}{MSE}=\overset{―}{VAR}+\overset{―}{BIAS^{2}}\\ \;=\frac{1}{N}\frac{1}{R}\sum_{i=1}^{N}\sum_{r=1}^{R}{\left({ê}_{\mathrm{ri}}-{\overset{―}{e}}_{\mathrm{Ri}}\right)}^{2}+\frac{1}{N}\sum_{i=1}^{N}{\left({\overset{―}{e}}_{\mathrm{Ri}}-e_{i}\right)}^{2}\text{.} \end{array}$$

In (3) $\overset{―}{VAR}$ is the average variance and $\overset{―}{BIAS^{2}}$ is the mean squared bias (MSB) over *N* simulations. It is noted that the two components in (3) are analogous to the pooled variance and lack-of-fit components in linear regression where there are *R* observations at each of *N* values of an independent variable.

With BCV, like *k*CV, it is possible to calculate the MSE in (1) for each value of the true conditional error (*k* <*n*). Each of the *R* bootstrap samples is drawn first, and then each of the *n* observations in the with-replacement sample is left out one at a time to get an estimate of the prediction error. With BT632, however, it is not possible to calculate the MSE in (1) because only one estimate of the true conditional error can be calculated from the *R* bootstrap samples in each of the *N* simulation runs. That is, with BT632 each of the *n* samples is left out one at a time and then *R* bootstrap samples are drawn from the remaining *n*-1 samples. These *R* bootstrap samples give an estimate of the prediction error for the left-out observation, and the average of these estimates over the *n* samples is the BT632 estimate. (Efron and Tibshirani [9] presented an efficient algorithm for computing BT632 that uses only *R* total bootstrap samples instead of *R* × *n* samples; the expected number of bootstrap samples used to estimate the prediction error for each left-out observation is (1–0.632) × *R*.) Hence, the decomposition in (3) can be achieved with both *k*CV and BCV, but not with BT632.

Of necessity, because of the construction of BT632 and associated estimators, Efron and Tibshirani [9] used only the MSB (second term in (3)) to evaluate the performance of cross-validation methods, where *ē*_*Ri*_ for BT632 has a different connotation than for *k*CV and BCV, but is still an average calculated from *R* (or fewer) bootstrap samples per observation. Similarly, Molinaro et al. [7] employed the MSB in their investigation. Although it was not explicitly shown, in both papers the MSB was further decomposed as

(4) $$\begin{array}{l} MSB={\left[SD\left(BIAS\right)\right]}^{2}+{\overset{―}{BIAS}}^{2}\\ =\frac{1}{N}\sum_{i=1}^{N}{\left\{\left({\overset{―}{e}}_{\mathrm{Ri}}-e_{i}\right)-\left({\overset{―}{e}}_{N}-\overset{―}{e}\right)\right\}}^{2}+{\left({\overset{―}{e}}_{N}-\overset{―}{e}\right)}^{2} \end{array}$$

where ${\overset{―}{e}}_{N}=\sum_{i=1}^{N}{\overset{―}{e}}_{\mathrm{Ri}}/N$ and $\overset{―}{e}=\sum_{i=1}^{N}e_{i}/N$, with interpretations of results based on the standard deviation of the bias, *SD*(*BIAS*), and the average bias, $\overset{―}{BIAS}$, but using different notation. Although the simulation study conducted by Fu et al. [10] provided information on the variance and bias components of the $\overset{―}{MSE}$ in (3) with respect to the BCV estimator of the true conditional error rate, the information on variance was not used in the comparison with LOOCV and BT632, as it was not possible to obtain equivalent information with the latter two methods. Instead, Fu et al. [10] presented information 2on components comparable to those of the MSB in (4), but defined on a relative basis. In the simulation study reported here, in addition to the information provided by the squared-bias component and its sub-components in (4), the information that both BCV and *k*CV provide on the variance component of the $\overset{―}{MSE}$ in (3) has been compared. To make the BCV-*k*CV comparison as fair as possible, the number of recomputations, i.e., the number of retrainings of a classifier, was equalized for BCV and *k*CV. The purpose was to equalize information rather than to equalize computational effort (see [9,11]). As with the study of Fu et al. [10], the present comparison was restricted to low-dimensional predictor spaces.

#### Monte Carlo simulation study

It was assumed that there were two populations (classes) defined by *p* ≥ 1 predictors or features having underlying Gaussian distributions [10]. The first population was assumed to be distributed *N*(**μ**_1_, **Σ**_1_) with **μ**_1_ = **0**_(*p*)_^'^ and the second *N*(**μ**_2_, **Σ**_2_) with $\mu_{2}={\left[\Delta_{\left(p\right)}/\sqrt{p}\right]}^{'}$, where **0**_(*p*)_ is the *p*-dimensional zero vector and **Δ**_(*p*)_ is a *p*-dimensional vector of non-zero constants, Δ. The structure of **μ**_2_ is a modified configuration of Freidman [12]. In addition to the equal variance case studied by Fu et al. [10], where **Σ**_1_ = **Σ**_2_ = **Ι**_(*p*)_ (the *p*×*p* identity matrix), here the case of unequal population variances was also studied, where **Σ**_1_ = **Ι**_(*p*)_ and **Σ**_2_ = 2**Ι**_(*p*)_. Independence among predictors, as reflected by **Σ**_1_ = **Ι**_(*p*)_ and **Σ**_2_ = 2**Ι**_(*p*)_, was assumed in order to be consistent with Fu et al. [10]. Given the mean structures, any positive correlation among predictors would simply decrease the generalized Mahalanobis distance between the two populations while negative correlation would increase the distance.

Feature dimensions of *p* = 1 and 5 were simulated, along with Δ = 1 and 3. For *p* = 1, sample sizes of *n* = 20, 50 and 100 were simulated (*n*/2 in each class), while for p = 5, only *n* = 50 and 100 were considered. Whereas Fu et al. [10] used quadratic discriminant analysis (QDA) to classify samples for some comparisons and a *k*-nearest neighbor (*k*-NN) classifier for others, here QDA was used for all comparisons in light of the low-dimensionality. For higher dimensions, where *p* >*n*, a method like *k*-NN would be required.

As mentioned above, there is only one way to do LOOCV with a given sample. On the other hand, BCV as defined by Fu et al. [10] uses an average of the LOOCV prediction errors over *B* bootstrap (re)samples. Hence, BCV is based on *B* × *n* recomputations (retrainings of the classifier) while LOOCV is based on only *n* recomputations. For a more extensive comparison, three approaches were taken here in order to compare BCV to *k*-fold CV (henceforward *k*CV), as summarized in Table 1.

**Table 1:** Cross-validation methods to be compared

**Method**	**No. Repetitions**	**Sample Type**	**Total No. Retrainings**
*k*CV*n*^1^	1	NA	*n*
BCV*n*^2^	*B*	Bootstrap	*B*×*n*
*k*CV*n*/2	2×*B*	Permutation	*B*×*n*
BCV*n*/2	2×*B*	Bootstrap	*B*×*n*
*k*CV10	*B*×*n*/10	Permutation	*B*×*n*
BCV10	*B*×*n*/10	Bootstrap	*B*×*n*

^1^*k*CV*n* is leave-one-out CV (LOOCV).

^2^BCV*n* is BCV as defined by Fu et al. (2005).

First, LOOCV was compared to BCV as was done by Fu and colleagues [10]. These methods are denoted by *k*CV*n* and BCV*n*, respectively, in Table 1. Second, *n*/2-fold CV (leave-two-out CV, denoted *k*CV*n*/2) was done in order to stay as close as possible to LOOCV (*k*CV*n*) while allowing multiple retrainings of the classifier. To keep the number of recomputations the same as for BCV, 2 × *B* repetitions of *k*CV*n*/2 were run (2×*B*×*n*/2 = *B* × *n*). Also, a version of BCV based on *n*/2-fold CV (BCV*n*/2) was implemented with 2 × *B* repetitions for a head-to-head comparison with *k*CV*n*/2 based on the same number, *B*×*n*, of total retrainings. Third, a version of BCV based on 10-fold CV (BCV10) was implemented and compared to traditional 10-fold CV (*k*CV10), where again the number of recomputations was the same. Here, both BCV10 and *k*CV10 were based on *B*×*n*/10 repetitions for a total of *B*×*n* retrainings. BCV*n*/2 and BCV10 were defined like *k*CV*n*/2 and *k*CV10, except that in each repetition, a bootstrap sample of size *n* was randomly divided into *n*/2 or 10 subsets, while with *k*CV*n*/2 and *k*CV10 the original *n* observations were randomly re-divided into *n*/2 or 10 subsets (these are the same when *n* = 20). In this study, *B* = 50 [9,10].

The simulation study was implemented as follows. For each combination of *p* and Δ, a “super-population” of size 10000 was drawn, 5000 from *N*(**μ**_1_, Σ _1_) and 5000 from *N*(μ_2_, **Σ** _2_). Then, for each value of *n*, *N* = 1000 simulations were run. For each simulation run, a stratified random sample of size *n* was drawn without replacement, *n*/2 observations from the 5000 *N*(**μ**_1_, **Σ** _1_) population values and *n*/2 observations from the 5000 *N*(μ_2_, Σ _2_) population values. The QDA classifier was trained on the sample. Following Molinaro et al. [7], the *true conditional error* rate for each classifier was calculated as the proportion of times the trained classifier misclassified the remaining 10000-*n* members of the super-population. Then *k*CV*n*, BCV*n*, *k*CV*n*/2, BCV*n*/2, *k*CV10 and BCV10 were each conducted on the sample to estimate the true conditional error. Their MSE, variance and bias were calculated from expression (1).

For *p*=1 it was found that at least four distinct observations were needed in each class to avoid numerical problems in training the QDA classifier for the BCV*n*/2 and BCV10 methods. Hence, this requirement was imposed on all three BCV methods. (Fu et al., [10], required at least three distinct observations in each class for the original BCV method, BCV*n*.) In addition, for *p*=5, the BCV methods were implemented with stratified sampling, i.e., *n*/2 bootstrap samples from each class, along with a requirement of at least eight distinct observations in each class.

The mean and standard deviation of the MSE, variance, and bias, as well as the MSB over the *N* = 1000 simulations were calculated for BCV and *k*CV. With *R* representing the number of repetitions of each method (Table 1, column 2), the means are defined by

(5) $$\overset{―}{MSE}=\left(1/N\right)\sum_{i=1}^{N}\left(1/R\right)\sum_{r=1}^{R}{\left({ê}_{\mathrm{ri}}-e_{i}\right)}^{2}\text{,}$$

(6) $$\overset{―}{VAR}=\left(1/N\right)\sum_{i=1}^{N}\left(1/R\right)\sum_{r=1}^{R}{\left({ê}_{\mathrm{ri}}-{\overset{―}{e}}_{\mathrm{Ri}}\right)}^{2}\text{,}$$

(7) $$\overset{―}{BIAS}=\left(1/N\right)\sum_{i=1}^{N}\left({\overset{―}{e}}_{\mathrm{Ri}}-e_{i}\right)\text{,}$$

(8) $$\overset{―}{BIAS^{2}}=\left(1/N\right)\sum_{i=1}^{N}{\left({\overset{―}{e}}_{\mathrm{Ri}}-e_{i}\right)}^{2}\text{.}$$

The three standard deviations for each method are defined by

(9) SD(MSE)=[1N-1∑i=1N{1/R∑R=1R(ȇi)2−MSE―2}]12

(10) $$SD(VAR)={\left[(1/\{N-1\})\sum_{i=1}^{N}{\left\{(1/R)\sum_{R=1}^{R}{\left({ê}_{\mathrm{ri}}-{\overset{―}{e}}_{\mathrm{ri}}\right)}^{2}-\overset{―}{VAR}\right\}}^{2}\right]}^{1/2}$$

(11) $$SD\left(BIAS\right)={\left[\left(1/\left\{N-1\right\}\right)\sum_{i=1}^{N}{\left\{\left({\overset{―}{e}}_{\mathrm{ri}}-e_{i}\right)-\overset{―}{BIAS}\right\}}^{2}\right]}^{1/2}$$

The means and standard deviations defined in (5) – (11) were used to compare the performance of the methods.

R Version 2.6.0 was used to conduct the Monte Carlo simulation study, with an independently written SAS/IML program being used to verify the mean calculations for the equal-variance case with *p*=1 [13,14].

### Results and discussion

**Figure 1:**
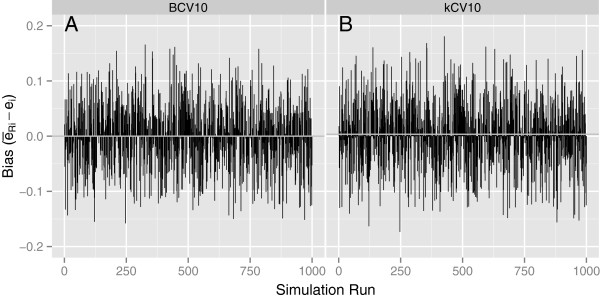
**The individual values of(*ē***_***Ri***_ **−** ***e***_***i***_**)that contribute to**$\overset{―}{BIAS}$**and *SD*(*BIAS*) for each of *N *= 1000 simulations with *p*=1, *n*=50,Δ = 1, and Σ**_**1**_ **=** **Σ**_**2**_ **=** **I.**

**Figure 2:**
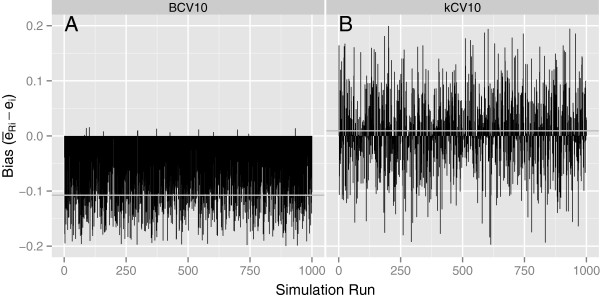
**The individual values of(*ē***_***Ri***_ **−** ***e***_***i***_**)that contribute to**$\overset{―}{BIAS}$**and *SD*(*BIAS*) for each of *N *= 1000 simulations with *p*=5, *n*=50,Δ = 1, and Σ**_**1**_ **=** **Σ**_**2**_ **=** **I.**

**Figure 3:**
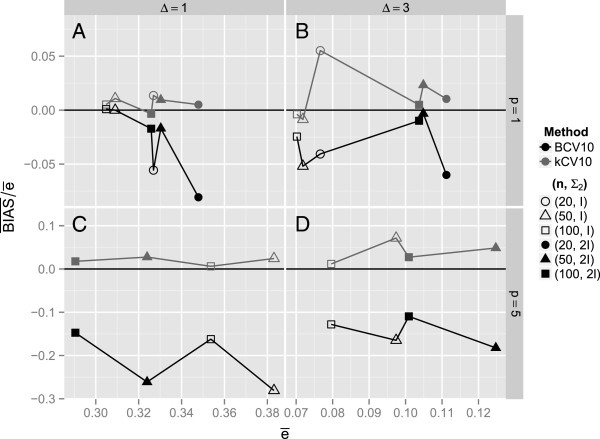
**The mean relative bias for each of the twenty simulation configurations in Tables**2**and**3**and inAdditional file**1**: Table S1, Additional file**2**: Table S2, Additional file**3**: Table S3 and Additional file**4**: Table S4, where each point is the average of *N *= 1000 values,**$\overset{―}{BIAS}/\overset{―}{e}\text{,}$**like those plotted in Figures**1**and**2 .

**Figure 4:**
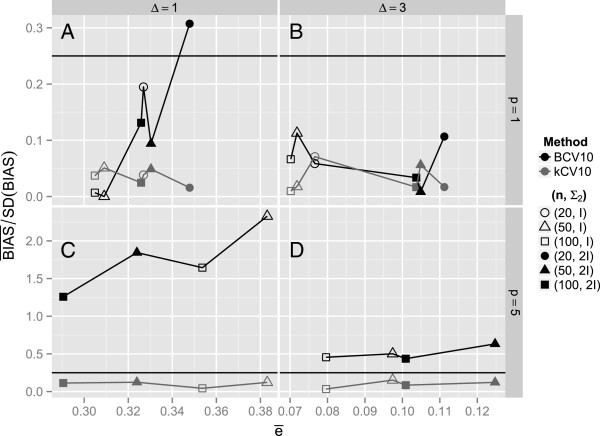
**The mean relative bias expressed as**$\overset{―}{BIAS}/SD\left(BIAS\right)$**for each of the twenty simulation configurations in Tables**2**and**3**and inAdditional file**1**: Table S1, Additional file**2**: Table S2, Additional file**3**: Table S3 and Additional file**4**: Table S4** .

The results of the simulation study are summarized in Tables 2 and 3 and Figures 1, 2, 3, 4. Table 2 is the same case as covered in Table 1 of Fu and colleagues [10]. For brevity, all configuration results are discussed but only a limited portion of the results are displayed in Tables 2 and 3 (i.e., cases for LOOCV, BCV*n*, *k*CV10, BCV10 where **Σ**_1_ = **Σ**_2_ = **Ι**_(*p*)_ ). The interested reader is referred to the supplementary material section for the tables in their entirety and for the cases where **Σ**_1_ = **Ι**_(*p*)_ and **Σ**_2_ = 2**Ι**_(*p*)_.

**Table 2:** **Simulation results for *p *= 1, Σ**_1_ = **Σ**_2_ = **I**_(1)_**, *N *= 1000**

**Method**	***n***	_**Δ**_	***ē***^**a**^	***ē***_***N***_	$\overset{―}{MSE}$	***SD*(*MSE*)**	$\overset{―}{VAR}$	***SD*(*VAR*)**	***MSB***	$\overset{―}{BIAS}$	***SD*(*BIAS*)**
LOOCV	50	1	0.30907	0.31124	0.00446	0.00635	0	0	0.00446	0.00217	0.06677
	50	3	0.07185	0.07114	0.00130	0.00184	0	0	0.00130	−0.00071	0.03603
BCV*n*	50	1	0.30907	0.30791	0.00942	0.00582	0.00543	0.00204	0.00399	−0.00116	0.06317
	50	3	0.07185	0.06781	0.00256	0.00180	0.00143	0.00074	0.00113	−0.00404	0.03340
LOOCV	100	1	0.30483	0.30579	0.00191	0.00281	0	0	0.00191	0.00096	0.04366
	100	3	0.07020	0.06987	0.00075	0.00114	0	0	0.00075	−0.00033	0.02741
BCV*n*	100	1	0.30483	0.30469	0.00419	0.00289	0.00243	0.00074	0.00175	−0.00013	0.04188
	100	3	0.07020	0.06829	0.00139	0.00109	0.00070	0.00030	0.00069	−0.00190	0.02621
*k*CV10	50	1	0.30907	0.31241	0.00461	0.00625	0.00029	0.00023	0.00432	0.00334	0.06568
	50	3	0.07185	0.07123	0.00131	0.00180	0.00008	0.00008	0.00123	−0.00062	0.03511
BCV10	50	1	0.30907	0.30906	0.00915	0.00526	0.00536	0.00145	0.00379	−0.00001	0.06163
	50	3	0.07185	0.06812	0.00252	0.00173	0.00141	0.00067	0.00111	−0.00373	0.03306
*k*CV10	100	1	0.30483	0.30642	0.00195	0.00280	0.00009	0.00005	0.00186	0.00160	0.04307
	100	3	0.07020	0.06993	0.00075	0.00108	0.00003	0.00002	0.00072	−0.00026	0.02683
BCV10	100	1	0.30483	0.30511	0.00411	0.00254	0.00241	0.00038	0.00170	0.00028	0.04125
	100	3	0.07020	0.06848	0.00136	0.00101	0.00069	0.00025	0.00067	−0.00171	0.02577

$\overset{―}{e}a=\left(1/N\right)\sum_{i=1}^{N}e_{i},\;{\overset{―}{e}}_{N}=\left(1/N\right)\sum_{i=1}^{N}{\overset{―}{e}}_{\mathrm{Ri}},\;MSB=\left(1/N\right)\sum_{i=1}^{N}{\left({\overset{―}{e}}_{\mathrm{Ri}}-e_{i}\right)}^{2}$

**Table 3:** **Simulation results for *p *= 5, Σ**_1_ = **Σ**_2_ = *Ι*_(5)_**, *N *= 1000**

**Method**	***n***	_**Δ**_	***ē***^**a**^	***ē***_***N***_	$\overset{―}{MSE}$	***SD*(*MSE*)**	$\overset{―}{VAR}$	***SD*(*VAR*)**	***MSB***	$\overset{―}{BIAS}$	***SD*(*BIAS*)**
LOOCV	50	1	0.38308	0.38848	0.00688	0.00996	0	0	0.00688	0.00540	0.08281
	50	3	0.09741	0.10052	0.00227	0.00338	0	0	0.00227	0.00311	0.04760
BCV*n*	50	1	0.38308	0.27058	0.02073	0.01027	0.00587	0.00162	0.01486	−0.11250	0.04693
	50	3	0.09741	0.07686	0.00305	0.00167	0.00160	0.00082	0.00145	−0.02055	0.03208
LOOCV	100	1	0.35366	0.35164	0.00291	0.00419	0	0	0.00291	−0.00202	0.05390
	100	3	0.07962	0.07906	0.00080	0.00110	0	0	0.00080	−0.00056	0.02822
BCV*n*	100	1	0.35366	0.29312	0.00799	0.00454	0.00308	0.00074	0.00491	−0.06054	0.03529
	100	3	0.07962	0.06781	0.00141	0.00080	0.00075	0.00031	0.00066	−0.01180	0.02278
*k*CV10	50	1	0.38308	0.39236	0.00711	0.00860	0.00126	0.00036	0.00585	0.00929	0.07598
	50	3	0.09741	0.10438	0.00252	0.00327	0.00039	0.00019	0.00213	0.00697	0.04568
BCV10	50	1	0.38308	0.27549	0.01950	0.00968	0.00579	0.00113	0.01371	−0.10758	0.04622
	50	3	0.09741	0.08135	0.00302	0.00163	0.00173	0.00078	0.00128	−0.01606	0.03202
*k*CV10	100	1	0.35366	0.35585	0.00299	0.00364	0.00049	0.00012	0.00251	0.00219	0.05003
	100	3	0.07962	0.08055	0.00085	0.00109	0.00010	0.00005	0.00075	0.00094	0.02735
BCV10	100	1	0.35366	0.29629	0.00756	0.00427	0.00305	0.00041	0.00451	−0.05737	0.03488
	100	3	0.07962	0.06942	0.00136	0.00072	0.00076	0.00026	0.00060	−0.01020	0.02239

$\overset{―}{e}a=\left(1/N\right)\sum_{i=1}^{N}e_{i},\;{\overset{―}{e}}_{N}=\left(1/N\right)\sum_{i=1}^{N}{\overset{―}{e}}_{\mathrm{Ri}},\;MSB=\left(1/N\right)\sum_{i=1}^{N}{({\overset{―}{e}}_{\mathrm{Ri}}-e_{i})}^{2}$

Beginning with the MSB (i.e., $\overset{―}{BIAS^{2}}$), which is the criterion used by Efron and Tibshirani [9], Molinaro et al. [7], Fu et al. [10] and Kim [11] to compare estimators of the true conditional classification error, it is shown in Table 2 that for *p* = 1 the MSB of *k*CV is always larger than that of BCV. In terms of the components of the MSB, this is due to a larger *SD*(*BIAS*) for *k*CV than BCV, although the $\overset{―}{BIAS}$ of BCV tends to be negative and is generally larger than that of *k*CV in absolute value for Δ = 3. These results are consistent for configurations with n=20 (see supplementary material, Additional file 1: Table S1 and Additional file 2: Table S2). For *p* = 5, the same pattern is shown in Table 3 for the MSB for Δ = 3, but the reverse is shown for Δ = 1, i.e., the MSB is larger for BCV, where the negative $\overset{―}{BIAS}$ of BCV is very pronounced. Thus, the variation of BCV, as measured by *SD*(*BIAS*) is indeed reduced compared to *k*CV, although a price is paid in terms of increased $\overset{―}{BIAS}$. Again, the results for equal and unequal covariance matrices are consistent (see supplementary material, Additional file 3: Table S3 and Additional file 4: Table S4).

The individual values of (*ē*_*Ri*_ − *e*_*i*_) that contribute to $\overset{―}{BIAS}$ and *SD*(*BIAS*) for two different simulation conditions are plotted in Figures 1 and 2 for each of N = 1000 simulations. Figures 1a and 1b are for BCV and *k*CV, respectively, for *p*=1, *n*=50 and Δ = 1, from Table 2. Figures 2a and 2b represent corresponding plots for *p*=5 from Table 3. Figure 1 represents one of the best configurations for BCV compared to *k*CV while Figure 2 represents one of the worst scenarios. As the figures show, the individual estimates of the true conditional error, *e*_*i*_, are extremely variable across the 1000 simulations. The variance of BCV is indeed less than that of *k*CV, but the negative bias of BCV can be substantial as the dimensionality of the feature space increases.

#### Why there is large variation in general

The large variation shown in Figures 1 and 2, along with the correspondingly large values of SD(BIAS) in Tables 2 and 3 for both kCV and BCV, are consistent with results of Efron and Tibshirani ([9], Tables Three to eight on pages 554-556) and Molinaro et al. ([7], Tables One and Four on pages 3304 and 3305), both of whom showed large standard deviations and, in some cases, large values of bias, for the classifiers and error estimation methods they studied. In fact, Efron and Tibshirani [9] noted that none of the methods correlates very well with the conditional error rate on a sample-by-sample basis. This lack of correlation in the present investigation, reflected by the large values of *SD*(*BIAS*), appears to be partly due to a problem with the way the true classification error is defined and estimated. As mentioned in the Introduction, the problem appears to be that the quantity purportedly being estimated, the true misclassification error of the trained classifier conditional on the training set, is defined as a single fixed quantity for a given set of data.

#### Possible alternative approach for estimation of SD(BIAS)

It does not seem logical to take the misclassification error as a fixed quantity and then use cross-validation to estimate it, because the true conditional error for any classifier trained using only part of the data within a cross-validation is not the same as the true conditional error of the classifier trained on the complete set of data, i.e., the quantity to be estimated. This leads to an inflated estimate of *SD*(*BIAS*). Although it might prove to be computationally prohibitive, it seems more logical to define and calculate a true conditional error for each training set within each of the *k* partitions of a cross-validation, say *e*_*ijr*_ (*i* = 1,…,*N*;*j* = 1,…,*k*;*r* = 1,…,*R*) and then obtain a corresponding estimate, ${\hat{e}}_{\mathrm{ijr}}$. Each difference, ${\hat{e}}_{\mathrm{ijr}}-e_{\mathrm{ijr}}$, would represent an estimate of the expected bias in estimating a true conditional error so defined. So, even though the conditional error itself would change from partition to partition, one could still obtain a sample of estimates of the bias in estimating such an error. The variation among these bias estimates would be expected to be less than that represented by *SD*(*BIAS*) in Tables 2 and 3 and reflected in Figures 1 and 2 with the customary method, because a source of variation heretofore not taken into account would be eliminated. This “more logical” approach provides insight into how the variation in the bias is artificially inflated when one attempts to use cross-validation to estimate a single, fixed “true conditional error” of a trained classifier. Attempting to estimate the elusive true conditional error is not recommended. Instead, a classifier’s generalization error in predicting future observations is the error that should be estimated, and is the error for which cross-validation is well-suited.

#### Average bias estimates Are representative

On the other hand, even though individual-run biases are likely overstated because of the inflated variance when defined in terms of a fixed true conditional error, nevertheless, the average bias, $\overset{―}{BIAS}$, calculated in the customary way ought to be representative of the average bias that would be reflected if the “more logical” method described above were used. For this reason, plots like Figure 3 of Efron and Tibshirani [9] of the average relative bias in terms of the *expected true error* are useful for comparing error estimation methods, even though the individual true conditional errors defined the usual way may not be estimated with precision. Figures 3a to 3d mimic Figure 3 of Efron and Tibshirani [9]. The plotted points are values of $\overset{―}{BIAS}/\overset{―}{e}\text{,}$ which is equivalent to (*ē*_*N*_ − *ē*)/*ē*, for each of the twenty simulation configurations in Tables 2 and 3 and supplementary material Additional file 1: Table S1, Additional file 2: Table S2, Additional file 3: Table S3 and Additional file 4: Table S4, where each point is the average of *N* = 1000 values like those plotted in Figures 1 and 2. For example, the open triangles plotted in Figure 3a and 3c correspond to Figures 1 and 2, respectively. These figures show a consistent, but modest, positive relative bias for *k*CV and a consistent, sometimes large, negative relative bias for BCV. In particular as shown in Figure 3, as the sample size increases, differences in relative bias between *k*CV and BCV decrease for both *p*=1 and p=5. This agrees with the result of a numerical experiment by Davison and Hall [15] with *p*=3 and *n*=(20, 40, 80) in a comparison of bootstrap and LOOCV estimates of discrimination error. Even so, the relative bias of BCV in Figure 3 for *p*=5 is still substantially negative when *n*=100. For *p*=3, Davison and Hall [15] observed a similar decrease in disagreement between the methods as the distance between populations increased. For *p*=5, Figure 3 also shows that effect going from Δ = 1 to Δ = 3.

#### Impact of BCV being negatively biased

The negative bias of BCV, i.e., underestimation of the true error, can be explained by the fact that the probability that a test sample appears in the training set is 1-(1-1/*n*)^*n*^ ≈ 0.632. Borrowing the words of Efron and Tibshirani [9] to describe this phenomenon, BCV “uses training samples that are too close to the test points, leading to potential underestimation of the error rate.” It is important to note that the bootstrap methods of Efron and Tibshirani do not include test points in the training set.

The substantial negative bias of BCV means that BCV tends to underestimate the classification error on average. While the direction and magnitude of the bias of a cross-validation method might not matter a great deal if the performances of several competitive classification procedures are being compared, it definitely matters if the error rate of a specific classification procedure is of interest. Substantial negative bias, translating to underestimation of the true misclassification error, would be a serious concern. To expound on the sizable negative bias of BCV, Figure 4 shows plots of $\overset{―}{BIAS}/SD\left(BIAS\right)$ for the same simulations as Figure 3. Applying the rule of thumb of Efron and Tibshirani [16] to bias estimation, the horizontal reference lines at 0.25 in each panel represent thresholds of acceptable relative bias. For *p*=1, both *k*CV and BCV satisfy the threshold, except for one instance where BCV exceeds the threshold slightly when *n*=20. However, for *p*=5, BCV always exceeds the threshold while *k*CV is always below the threshold. When Δ = 1, all four BCV relative biases exceed 1, i.e., they are more than four times the 0.25 threshold. Alternatively, a relative-bias plot could be constructed using averages of the components of the MSE by plotting the ratio $\overset{―}{BIAS}/\sqrt{\overset{―}{VAR}}$. This would show the same general result as Figure 4, but would be less pronounced because of the propensity for *increased*$\overset{―}{VAR}$ of BCV compared to *k*CV for estimating individual *e*(*e*_*i*_ = 1,…,*N*) (Tables 2, 3) due to the positive covariance induced by with-replacement sampling with the BCV method.

#### Assessing reproducibility of error estimates

Because both BCV and *k*CV can be repeated multiple times, as they have been in the present simulation study, they can give information on the reproducibility among repeated cross-validations. The values of $\overset{―}{MSE}$, *SD*(*MSE*), $\overset{―}{VAR}$ and *SD*(*VAR*) in Tables 2 and 3 provide such information on the reproducibility of BCV and *k*CV from CV-run to CV-run. When *k*CV is used in practice, where there is only a single set of training data, either *VAR* or $\sqrt{VAR}$ is the commonly reported value along with the average error, ${\overset{―}{e}}_{R}=\sum_{r=1}^{R}{ê}_{r}/N$, or its complement, the average accuracy [1,5]. Because the purpose of cross-validation is to assess a classifier’s ability to generalize outside the training set, the variation from CV-run to CV-run is an important measure of performance. Note that even though the present problem may be ill-defined such that the average biases for individual simulation runs are exaggerated, the values of $\overset{―}{VAR}$ are unaffected and correctly reflect the degree of reproducibility of the generalization error estimate.

#### Fair comparison requires equalization of number of trainings

In this study, there were 100 to 500 repetitions of each method (1000 to 5000 retrainings) in order to put BCV and *k*CV on the same footing with respect to the number of retrainings of classifiers [9,11]. This is many more repetitions than the ten or twenty repetitions normally done with *k*CV10. Although nowadays CPU time is relatively inexpensive, 100 to 500 repetitions may be excessive. On the other hand, although the BT632 method of Efron and Tibshirani [9] did not perform as well overall as BCV in the study of Fu et al. [10], it did show competitive behavior in some cases. It seems likely that, if the number of retrainings were equalized while employing the economical algorithm of Efron and Tibshirani [9], the competitiveness of BT632 evaluated in terms of average squared bias and its component parts would improve. As Kim [11] reported recently, the BT632^+^ method based on 50 bootstraps performed better than 5 repetitions of *k*CV10 in terms of average squared bias for a pruned tree classifier, although it, too, had a downward bias.

#### Microarray example

We evaluated the performance of *k*CV and BCV in predicting prognosis based on the gene expression profiles of breast cancer patients previously reported by van’t Veer and colleagues [17,18]. The van de Vijver *et al*. [17] study consisted of 295 patients with stage I or II breast cancer, and patients’ prognosis and gene expression data are publicly available at http://microarray-pubs.stanford.edu/would_NKI/explore.html. While the dataset contained a 70-gene prognosis profile, we chose to perform our evaluation of *k*CV and BCV using only 5 genes based on a simple gene selection procedure using a t-statistic with adjusted p-values [19]. In the study of Fu et al. [10], the authors chose 5 genes that were most highly correlated with the patient’s prognosis. As noted in Fu et al. [10], such gene selection procedure is prone to bias. However, the purpose of this evaluation is not gene selection. Furthermore, in practice only a small subset of genes is often of clinical interest.

Following the steps taken in Fu et al. [10] for comparison, the subsequent steps were carried out: (1) take a random sample *S* of size *n* = 50 with half of the patients having good prognosis and half having poor prognosis, (2) train a QDA classifier based on the random sample *S* and compute its *true conditional error* rate based on the proportion of times the trained classifier misclassified the remaining samples, (3) for each random sample *S*, estimate the true conditional error for LOOCV, CVn/2, CV10, BCV, BCVn/2, and BCV10, (4) calculate their MSE, variance and bias, (5) repeat over 1000 simulation runs and calculate the mean and standard deviation of the MSE, variance, bias and MSB.

**Figure 5:**
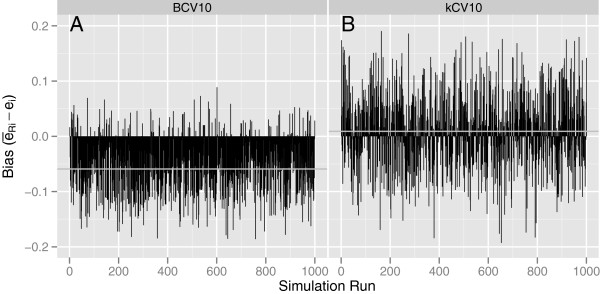
**The individual values of(*ē***_***Ri***_ **−** ***e***_***i***_**)that contribute to**$\overset{―}{BIAS}$**and *SD*(*BIAS*) for each of *N *= 1000 simulation runs using the breast cancer data.**

The results presented in Table 4 and Figure 5 are consistent with our simulation results in Table 3 for Δ = 1. More specifically, the MSB is larger for BCV and the negative $\overset{―}{BIAS}$ of BCV is evident. Figure 5 certainly demonstrates that BCV is less variable, but as previously noted, this advantage is negated by the considerable bias and overall MSB. Furthermore, given this microarray example data, the $\overset{―}{MSE}$ and $\overset{―}{VAR}$ for the BCV methods are higher than the corresponding quantities for the *k*CV counterpart.

**Table 4:** Results for the microarray example

**Method**	***n***	***ē***^**a**^	***ē***_***N***_	$\overset{―}{MSE}$	***SD*(*MSE*)**	$\overset{―}{VAR}$	***SD*(*VAR*)**	***MSB***	$\overset{―}{BIAS}$	***SD*(*BIAS*)**
LOOCV	50	0.2363	0.2404	0.0056	0.0077	0	0	0.0056	0.0042	0.0746
BCV*n*	50	0.2363	0.1726	0.0104	0.0070	0.0037	0.0013	0.0067	−0.0636	0.0517
*k*CV*n*/2	50	0.2363	0.2418	0.0056	0.0074	0.0003	0.0001	0.0053	0.0055	0.0729
BCV*n*/2	50	0.2363	0.1736	0.0102	0.0069	0.0037	0.0011	0.0065	−0.0627	0.0511
*k*CV10	50	0.2363	0.2454	0.0057	0.0070	0.0007	0.0003	0.0050	0.0091	0.0704
BCV10	50	0.2363	0.1772	0.0098	0.0066	0.0037	0.0010	0.0061	−0.0591	0.0508

$\overset{―}{e}a=\left(1/N\right)\sum_{i=1}^{N}e_{i},\;{\overset{―}{e}}_{N}=\left(1/N\right)\sum_{i=1}^{N}{\overset{―}{e}}_{\mathrm{Ri}},\;MSB=\left(1/N\right)\sum_{i=1}^{N}{({\overset{―}{e}}_{\mathrm{Ri}}-e_{i})}^{2}.$

## Conclusions

Cross-validation is a widely accepted and sound practice for estimating the *generalization error* of a classifier. Of course, for small data sets with high-dimensional predictors, especially for *p* >*n*, the variation among cross-validated error estimates can be large. For methods like BCV and kCV that can be replicated, it is generally accepted that cross-validation should be repeated 10 to 30 times to account for variation. However, using cross-validation to estimate the *fixed misclassification error* of a trained classifier conditional on the training set is problematic and should not be attempted. Although Monte Carlo simulation of this estimation exercise can correctly represent the average bias, it will overstate the variance of the bias. For the low-dimensional conditions simulated in the present study, *k*CV showed a consistent, but modest, positive bias. Conversely, BCV showed a consistent, and sometimes substantial, negative bias, which was much more pronounced for *p*=5 than for *p*=1. Increasing the complexity of the simulation to incorporate higher dimensions would only magnify the effect. The bias of BCV is too high a price to pay for its reduced variance; *k*-fold CV is recommended.

## Abbreviations

CV: Cross-validation; MSE: Mean squared error; MSB: Mean squared bias; BCV: Bootstrap cross-validation; LOOCV: Leave-one-out cross-validation; RMS: Root mean square; MSRE: Mean squared relative error; *k*CV: k-fold cross-validation; QDA: quadratic discriminant analysis; *k*NN: *k*-nearest neighbor.

## Competing interests

The authors declare that they have no competing interests relevant to this article to disclose.

## Author’s contributions

SO and RLK conceived the problem and designed the simulations for the manuscript. SYL and HJS were in charge of the computational coding. All authors were involved in drafting the manuscript. All authors read and approved the final manuscript.

## Authors’ information

Songthip Ounpraseuth is an Associate Professor in Department of Biostatistics at the University of Arkansas for Medical Science, Little Rock, AR. His research interests include prediction error estimation, computational statistics, dimension reduction and classification.

Ralph L Kodell is a Professor in the Department of Biostatistics at the University of Arkansas for Medical Sciences. His research interests include classification algorithms for biomedical decision making and statistical models and methods for toxicology and risk assessment.

Shelly Y. Lensing is a Research Associate biostatistician in the Department of Biostatistics at the University of Arkansas for Medical Sciences. Her research interests are the design and analysis of clinical trials and statistical computing.

Horace J. Spencer is a Research Associate biostatistician in the Department of Biostatistics at the University of Arkansas for Medical Sciences. His research interests are in all aspects of statistical computing and error estimation.

## Financial disclosures

The authors have no financial relationships relevant to this article to disclose.

## Supplementary Material

Additional file 1 Table S1 Simulation results for *p* = 1, ∑_1_ = ∑_2_ = I_(1)_, *N* = 1000. Click here for file

Additional file 2 Table S2 Simulation results for *p* = 5, ∑_1_ = ∑_2_ = I_(5)_, *N* = 1000. Click here for file

Additional file 3 Table S3 Simulation results for *p* = 1, ∑_1_ = *I*_(1)_,∑_2_ = 2*I*,*N* = 1000. Click here for file

Additional file 4 Table S4 Simulation results for *p* = 5, ∑_1_ = *I*_(5)_,∑_2_ = 2*I*_(5)_,*N* = 1000. Click here for file
