# Supplementary material for: Estimating misclassification error: a closer look at cross-validation based methods
Source: BMC Res Notes. 2012 Nov 28;5:656. doi: 10.1186/1756-0500-5-656 (PMC3556102; doi:10.1186/1756-0500-5-656)
Supplement: Additional file 1 Table S1 — Simulation results for p = 1, ∑1 = ∑2 = I(1), N = 1000. [file 1756-0500-5-656-S1.doc]

Table S1. Simulation results for *p* = 1, $\mathbf{\Sigma}_{1}=\mathbf{\Sigma}_{2}=\mathbf{I}_{(1)}$, *N* = 1000.

| Method | *n* | $\Delta$ | $\bar{e}$^a^ | $\bar{e}_{N}$ | $\overline{MSE}$ | $SD(MSE)$ | $\overline{VAR}$ | $SD(VAR)$ | | $MSB$ | | $\overline{BIAS}$ | | $SD(BIAS)$ | |
| --- | --- | --- | --- | --- | --- | --- | --- | --- | --- | --- | --- | --- | --- | --- | --- |
|  |  |  |  |  |  |  |  |  | |  | |  | |  | |
| LOOCV | 20 | 1 | 0.32691 | 0.32995 | 0.01441 | 0.03000 | 0 | | 0 | | 0.01441 | 0.00304 | 0.12007 | |  |
|  | 20 | 3 | 0.07662 | 0.08060 | 0.00389 | 0.00689 | 0 | | 0 | | 0.00389 | 0.00398 | 0.06226 | |  |
|  | 50 | 1 | 0.30907 | 0.31124 | 0.00446 | 0.00635 | 0 | | 0 | | 0.00446 | 0.00217 | 0.06677 | |  |
|  | 50 | 3 | 0.07185 | 0.07114 | 0.00130 | 0.00184 | 0 | | 0 | | 0.00130 | -0.00071 | 0.03603 | |  |
|  | 100 | 1 | 0.30483 | 0.30579 | 0.00191 | 0.00281 | 0 | | 0 | | 0.00191 | 0.00096 | 0.04366 | |  |
|  | 100 | 3 | 0.07020 | 0.06987 | 0.00075 | 0.00114 | 0 | | 0 | | 0.00075 | -0.00033 | 0.02741 | |  |
| BCV*n* | 20 | 1 | 0.32691 | 0.30658 | 0.02490 | 0.01301 | 0.01589 | | 0.00725 | | 0.00901 | -0.02033 | 0.09279 | |  |
|  | 20 | 3 | 0.07662 | 0.07254 | 0.00659 | 0.00619 | 0.00372 | | 0.00294 | | 0.00288 | -0.00408 | 0.05350 | |  |
|  | 50 | 1 | 0.30907 | 0.30791 | 0.00942 | 0.00582 | 0.00543 | | 0.00204 | | 0.00399 | -0.00116 | 0.06317 | |  |
|  | 50 | 3 | 0.07185 | 0.06781 | 0.00256 | 0.00180 | 0.00143 | | 0.00074 | | 0.00113 | -0.00404 | 0.03340 | |  |
|  | 100 | 1 | 0.30483 | 0.30469 | 0.00419 | 0.00289 | 0.00243 | | 0.00074 | | 0.00175 | -0.00013 | 0.04188 | |  |
|  | 100 | 3 | 0.07020 | 0.06829 | 0.00139 | 0.00109 | 0.00070 | | 0.00030 | | 0.00069 | -0.00190 | 0.02621 | |  |
|  |  |  |  |  |  |  |  | |  | |  |  |  | |  |
| *k*CV*n*/2 | 20 | 1 | 0.32691 | 0.33136 | 0.01465 | 0.02389 | 0.00119 | | 0.00123 | | 0.01347 | 0.00445 | 0.11601 | |  |
|  | 20 | 3 | 0.07662 | 0.08084 | 0.00389 | 0.00592 | 0.00032 | | 0.00037 | | 0.00357 | 0.00422 | 0.05962 | |  |
|  | 50 | 1 | 0.30907 | 0.31153 | 0.00448 | 0.00620 | 0.00012 | | 0.00012 | | 0.00436 | 0.00246 | 0.06604 | |  |
|  | 50 | 3 | 0.07185 | 0.07111 | 0.00130 | 0.00183 | 0.00003 | | 0.00005 | | 0.00126 | -0.00074 | 0.03557 | |  |
|  | 100 | 1 | 0.30483 | 0.30606 | 0.00192 | 0.00285 | 0.00003 | | 0.00002 | | 0.00190 | 0.00123 | 0.04355 | |  |
|  | 100 | 3 | 0.07020 | 0.06985 | 0.00075 | 0.00113 | 0.00001 | | 0.00001 | | 0.00074 | -0.00035 | 0.02722 | |  |
| BCV*n*/2 | 20 | 1 | 0.32691 | 0.30753 | 0.02412 | 0.01169 | 0.01530 | | 0.00590 | | 0.00882 | -0.01938 | 0.09194 | |  |
|  | 20 | 3 | 0.07662 | 0.07361 | 0.00654 | 0.00618 | 0.00373 | | 0.00274 | | 0.00281 | -0.00302 | 0.05299 | |  |
|  | 50 | 1 | 0.30907 | 0.30806 | 0.00920 | 0.00549 | 0.00535 | | 0.00166 | | 0.00385 | -0.00101 | 0.06211 | |  |
|  | 50 | 3 | 0.07185 | 0.06798 | 0.00255 | 0.00173 | 0.00143 | | 0.00069 | | 0.00112 | -0.00387 | 0.03323 | |  |
|  | 100 | 1 | 0.30483 | 0.30464 | 0.00412 | 0.00257 | 0.00241 | | 0.00052 | | 0.00171 | -0.00019 | 0.04137 | |  |
|  | 100 | 3 | 0.07020 | 0.06835 | 0.00137 | 0.00104 | 0.00069 | | 0.00027 | | 0.00068 | -0.00185 | 0.02595 | |  |
|  |  |  |  |  |  |  |  | |  | |  |  |  | |  |
| *k*CV10 | 20 | 1 | 0.32691 | 0.33138 | 0.01466 | 0.02390 | 0.00118 | | 0.00120 | | 0.01348 | 0.00447 | 0.11609 | |  |
|  | 20 | 3 | 0.07662 | 0.08085 | 0.00390 | 0.00594 | 0.00032 | | 0.00037 | | 0.00358 | 0.00423 | 0.05967 | |  |
|  | 50 | 1 | 0.30907 | 0.31241 | 0.00461 | 0.00625 | 0.00029 | | 0.00023 | | 0.00432 | 0.00334 | 0.06568 | |  |
|  | 50 | 3 | 0.07185 | 0.07123 | 0.00131 | 0.00180 | 0.00008 | | 0.00008 | | 0.00123 | -0.00062 | 0.03511 | |  |
|  | 100 | 1 | 0.30483 | 0.30642 | 0.00195 | 0.00280 | 0.00009 | | 0.00005 | | 0.00186 | 0.00160 | 0.04307 | |  |
|  | 100 | 3 | 0.07020 | 0.06993 | 0.00075 | 0.00108 | 0.00003 | | 0.00002 | | 0.00072 | -0.00026 | 0.02683 | |  |
| BCV10 | 20 | 1 | 0.32691 | 0.30875 | 0.02431 | 0.01190 | 0.01531 | | 0.00595 | | 0.00900 | -0.01816 | 0.09315 | |  |
|  | 20 | 3 | 0.07662 | 0.07351 | 0.00655 | 0.00611 | 0.00374 | | 0.00269 | | 0.00282 | -0.00311 | 0.05301 | |  |
|  | 50 | 1 | 0.30907 | 0.30906 | 0.00915 | 0.00526 | 0.00536 | | 0.00145 | | 0.00379 | -0.00001 | 0.06163 | |  |
|  | 50 | 3 | 0.07185 | 0.06812 | 0.00252 | 0.00173 | 0.00141 | | 0.00067 | | 0.00111 | -0.00373 | 0.03306 | |  |
|  | 100 | 1 | 0.30483 | 0.30511 | 0.00411 | 0.00254 | 0.00241 | | 0.00038 | | 0.00170 | 0.00028 | 0.04125 | |  |
|  | 100 | 3 | 0.07020 | 0.06848 | 0.00136 | 0.00101 | 0.00069 | | 0.00025 | | 0.00067 | -0.00171 | 0.02577 | |  |

$${}^{a}\bar{e}=(1/N)\sum_{i=1}^{N} e_{i}, \bar{e}_{N}=(1/N)\sum_{i=1}^{N} \bar{e}_{Ri}, \overline{BIAS^{2}}=(1/N)\sum_{i=1}^{N} (\bar{e}_{Ri}-e_{i})^{2}$$
