# Supplementary material for: Estimating misclassification error: a closer look at cross-validation based methods
Source: BMC Res Notes. 2012 Nov 28;5:656. doi: 10.1186/1756-0500-5-656 (PMC3556102; doi:10.1186/1756-0500-5-656)
Supplement: Additional file 2 Table S2 — Simulation results for p = 5, ∑1 = ∑2 = I(5), N = 1000. [file 1756-0500-5-656-S2.doc]

Table S2. Simulation results for *p* = 5, $\mathbf{\Sigma}_{1}=\mathbf{\Sigma}_{2}=I_{(5)}$, *N* = 1000.

| Method | *n* | $\Delta$ | $\bar{e}$^a^ | $\bar{e}_{N}$ | $\overline{MSE}$ | $SD(MSE)$ | $\overline{VAR}$ | $SD(VAR)$ | $MSB$ | $\overline{BIAS}$ | $SD(BIAS)$ |
| --- | --- | --- | --- | --- | --- | --- | --- | --- | --- | --- | --- |

| LOOCV | 50 | 1 | 0.38308 | 0.38848 | 0.00688 | 0.00996 | 0 | 0 | 0.00688 | 0.00540 | 0.08281 |
| --- | --- | --- | --- | --- | --- | --- | --- | --- | --- | --- | --- |
|  | 50 | 3 | 0.09741 | 0.10052 | 0.00227 | 0.00338 | 0 | 0 | 0.00227 | 0.00311 | 0.04760 |
|  | 100 | 1 | 0.35366 | 0.35164 | 0.00291 | 0.00419 | 0 | 0 | 0.00291 | -0.00202 | 0.05390 |
|  | 100 | 3 | 0.07962 | 0.07906 | 0.00080 | 0.00110 | 0 | 0 | 0.00080 | -0.00056 | 0.02822 |
| BCV*n* | 50 | 1 | 0.38308 | 0.27058 | 0.02073 | 0.01027 | 0.00587 | 0.00162 | 0.01486 | -0.11250 | 0.04693 |
|  | 50 | 3 | 0.09741 | 0.07686 | 0.00305 | 0.00167 | 0.00160 | 0.00082 | 0.00145 | -0.02055 | 0.03208 |
|  | 100 | 1 | 0.35366 | 0.29312 | 0.00799 | 0.00454 | 0.00308 | 0.00074 | 0.00491 | -0.06054 | 0.03529 |
|  | 100 | 3 | 0.07962 | 0.06781 | 0.00141 | 0.00080 | 0.00075 | 0.00031 | 0.00066 | -0.01180 | 0.02278 |
|  |  |  |  |  |  |  |  |  |  |  |  |
| *k*CV*n*/2 | 50 | 1 | 0.38308 | 0.38903 | 0.00691 | 0.00937 | 0.00053 | 0.00022 | 0.00638 | 0.00595 | 0.07971 |
|  | 50 | 3 | 0.09741 | 0.10103 | 0.00233 | 0.00326 | 0.00015 | 0.00010 | 0.00218 | 0.00362 | 0.04658 |
|  | 100 | 1 | 0.35366 | 0.35214 | 0.00290 | 0.00399 | 0.00013 | 0.00005 | 0.00277 | -0.00152 | 0.05263 |
|  | 100 | 3 | 0.07962 | 0.07909 | 0.00081 | 0.00110 | 0.00003 | 0.00002 | 0.00078 | -0.00053 | 0.02792 |
| BCV*n*/2 | 50 | 1 | 0.38308 | 0.27145 | 0.02043 | 0.01030 | 0.00576 | 0.00127 | 0.01467 | -0.11162 | 0.04704 |
|  | 50 | 3 | 0.09741 | 0.07800 | 0.00303 | 0.00167 | 0.00162 | 0.00081 | 0.00140 | -0.01941 | 0.03206 |
|  | 100 | 1 | 0.35366 | 0.29344 | 0.00795 | 0.00450 | 0.00308 | 0.00062 | 0.00488 | -0.06022 | 0.03537 |
|  | 100 | 3 | 0.07962 | 0.06800 | 0.00138 | 0.00074 | 0.00075 | 0.00028 | 0.00064 | -0.01161 | 0.02238 |
|  |  |  |  |  |  |  |  |  |  |  |  |
| *k*CV10 | 50 | 1 | 0.38308 | 0.39236 | 0.00711 | 0.00860 | 0.00126 | 0.00036 | 0.00585 | 0.00929 | 0.07598 |
|  | 50 | 3 | 0.09741 | 0.10438 | 0.00252 | 0.00327 | 0.00039 | 0.00019 | 0.00213 | 0.00697 | 0.04568 |
|  | 100 | 1 | 0.35366 | 0.35585 | 0.00299 | 0.00364 | 0.00049 | 0.00012 | 0.00251 | 0.00219 | 0.05003 |
|  | 100 | 3 | 0.07962 | 0.08055 | 0.00085 | 0.00109 | 0.00010 | 0.00005 | 0.00075 | 0.00094 | 0.02735 |
| BCV10 | 50 | 1 | 0.38308 | 0.27549 | 0.01950 | 0.00968 | 0.00579 | 0.00113 | 0.01371 | -0.10758 | 0.04622 |
|  | 50 | 3 | 0.09741 | 0.08135 | 0.00302 | 0.00163 | 0.00173 | 0.00078 | 0.00128 | -0.01606 | 0.03202 |
|  | 100 | 1 | 0.35366 | 0.29629 | 0.00756 | 0.00427 | 0.00305 | 0.00041 | 0.00451 | -0.05737 | 0.03488 |
|  | 100 | 3 | 0.07962 | 0.06942 | 0.00136 | 0.00072 | 0.00076 | 0.00026 | 0.00060 | -0.01020 | 0.02239 |

$${}^{a}\bar{e}=(1/N)\sum_{i=1}^{N} e_{i}, \bar{e}_{N}=(1/N)\sum_{i=1}^{N} \bar{e}_{Ri}, \overline{BIAS^{2}}=(1/N)\sum_{i=1}^{N} (\bar{e}_{Ri}-e_{i})^{2}$$
