# Supplementary material for: Estimating misclassification error: a closer look at cross-validation based methods
Source: BMC Res Notes. 2012 Nov 28;5:656. doi: 10.1186/1756-0500-5-656 (PMC3556102; doi:10.1186/1756-0500-5-656)
Supplement: Additional file 3 Table S3 — Simulation results for p = 1, ∑1 = I(1),∑2 = 2I,N = 1000. [file 1756-0500-5-656-S3.doc]

Table S3. Simulation results for *p* = 1, $\mathbf{\Sigma}_{1}=I_{(1)},\mathbf{\Sigma}_{2}=2I_{(1)}$, *N* = 1000.

| Method | *n* | $\Delta$ | $\bar{e}$^a^ | $\bar{e}_{N}$ | $\overline{MSE}$ | $SD(MSE)$ | $\overline{VAR}$ | $SD(VAR)$ | $MSB$ | $\overline{BIAS}$ | $SD(BIAS)$ |
| --- | --- | --- | --- | --- | --- | --- | --- | --- | --- | --- | --- |

| LOOCV | 20 | 1 | 0.34787 | 0.34795 | 0.01343 | 0.01972 | 0 | 0 | 0.01343 | 0.00008 | 0.11595 |
| --- | --- | --- | --- | --- | --- | --- | --- | --- | --- | --- | --- |
|  | 20 | 3 | 0.11120 | 0.11165 | 0.00497 | 0.00740 | 0 | 0 | 0.00497 | 0.00045 | 0.07051 |
|  | 50 | 1 | 0.33032 | 0.33252 | 0.00440 | 0.00659 | 0 | 0 | 0.00440 | 0.00220 | 0.06630 |
|  | 50 | 3 | 0.10488 | 0.10686 | 0.00201 | 0.00282 | 0 | 0 | 0.00201 | 0.00198 | 0.04483 |
|  | 100 | 1 | 0.32584 | 0.32402 | 0.00211 | 0.00287 | 0 | 0 | 0.00211 | -0.00182 | 0.04590 |
|  | 100 | 3 | 0.10371 | 0.10393 | 0.00101 | 0.00144 | 0 | 0 | 0.00101 | 0.00022 | 0.03176 |
| BCV*n* | 20 | 1 | 0.34787 | 0.31869 | 0.02575 | 0.01288 | 0.01639 | 0.00763 | 0.00936 | -0.02918 | 0.09226 |
|  | 20 | 3 | 0.11120 | 0.10247 | 0.00925 | 0.00685 | 0.00526 | 0.00342 | 0.00399 | -0.00874 | 0.06261 |
|  | 50 | 1 | 0.33032 | 0.32392 | 0.00936 | 0.00567 | 0.00568 | 0.00206 | 0.00368 | -0.00640 | 0.06038 |
|  | 50 | 3 | 0.10488 | 0.10433 | 0.00381 | 0.00259 | 0.00207 | 0.00087 | 0.00173 | -0.00054 | 0.04162 |
|  | 100 | 1 | 0.32584 | 0.32033 | 0.00439 | 0.00251 | 0.00250 | 0.00068 | 0.00188 | -0.00551 | 0.04308 |
|  | 100 | 3 | 0.10371 | 0.10250 | 0.00191 | 0.00134 | 0.00100 | 0.00035 | 0.00091 | -0.00120 | 0.03022 |
|  |  |  |  |  |  |  |  |  |  |  |  |
| *k*CV*n*/2 | 20 | 1 | 0.34787 | 0.34980 | 0.01395 | 0.01900 | 0.00126 | 0.00115 | 0.01268 | 0.00194 | 0.11266 |
|  | 20 | 3 | 0.11120 | 0.11236 | 0.00506 | 0.00698 | 0.00037 | 0.00039 | 0.00469 | 0.00116 | 0.06852 |
|  | 50 | 1 | 0.33031 | 0.33279 | 0.00443 | 0.00635 | 0.00015 | 0.00014 | 0.00428 | 0.00248 | 0.06542 |
|  | 50 | 3 | 0.10488 | 0.10713 | 0.00200 | 0.00276 | 0.00004 | 0.00006 | 0.00195 | 0.00226 | 0.04418 |
|  | 100 | 1 | 0.32584 | 0.32427 | 0.00213 | 0.00285 | 0.00003 | 0.00002 | 0.00209 | -0.00157 | 0.04575 |
|  | 100 | 3 | 0.10371 | 0.10404 | 0.00101 | 0.00142 | 0.00001 | 0.00001 | 0.00099 | 0.00033 | 0.03155 |
| BCV*n*/2 | 20 | 1 | 0.34787 | 0.31979 | 0.02492 | 0.01168 | 0.01572 | 0.00589 | 0.00920 | -0.02808 | 0.09176 |
|  | 20 | 3 | 0.11120 | 0.10420 | 0.00916 | 0.00647 | 0.00525 | 0.00321 | 0.00391 | -0.00701 | 0.06216 |
|  | 50 | 1 | 0.33032 | 0.32384 | 0.00920 | 0.00521 | 0.00560 | 0.00167 | 0.00360 | -0.00647 | 0.05970 |
|  | 50 | 3 | 0.10488 | 0.10440 | 0.00376 | 0.00250 | 0.00206 | 0.00080 | 0.00170 | -0.00047 | 0.04126 |
|  | 100 | 1 | 0.32584 | 0.31993 | 0.00437 | 0.00253 | 0.00251 | 0.00050 | 0.00187 | -0.00591 | 0.04281 |
|  | 100 | 3 | 0.10371 | 0.10249 | 0.00191 | 0.00130 | 0.00100 | 0.00031 | 0.00091 | -0.00121 | 0.03013 |
|  |  |  |  |  |  |  |  |  |  |  |  |
| *k*CV10 | 20 | 1 | 0.34787 | 0.34965 | 0.01394 | 0.01906 | 0.00125 | 0.00114 | 0.01269 | 0.00178 | 0.11271 |
|  | 20 | 3 | 0.11120 | 0.11236 | 0.00505 | 0.00698 | 0.00036 | 0.00038 | 0.00469 | 0.00116 | 0.06851 |
|  | 50 | 1 | 0.33032 | 0.33346 | 0.00454 | 0.00624 | 0.00035 | 0.00026 | 0.00418 | 0.00315 | 0.06464 |
|  | 50 | 3 | 0.10488 | 0.10732 | 0.00200 | 0.00269 | 0.00010 | 0.00008 | 0.00190 | 0.00244 | 0.04351 |
|  | 100 | 1 | 0.32584 | 0.32473 | 0.00216 | 0.00277 | 0.00012 | 0.00006 | 0.00204 | -0.00111 | 0.04517 |
|  | 100 | 3 | 0.10371 | 0.10422 | 0.00101 | 0.00139 | 0.00004 | 0.00003 | 0.00098 | 0.00052 | 0.03125 |
| BCV10 | 20 | 1 | 0.34787 | 0.31981 | 0.02486 | 0.01148 | 0.01575 | 0.00580 | 0.00911 | -0.02806 | 0.09126 |
|  | 20 | 3 | 0.11120 | 0.10453 | 0.00914 | 0.00649 | 0.00520 | 0.00310 | 0.00394 | -0.00667 | 0.06244 |
|  | 50 | 1 | 0.33032 | 0.32474 | 0.00913 | 0.00516 | 0.00559 | 0.00146 | 0.00355 | -0.00558 | 0.05931 |
|  | 50 | 3 | 0.10488 | 0.10454 | 0.00370 | 0.00244 | 0.00204 | 0.00075 | 0.00166 | -0.00034 | 0.04080 |
|  | 100 | 1 | 0.32584 | 0.32024 | 0.00434 | 0.00247 | 0.00248 | 0.00039 | 0.00185 | -0.00560 | 0.04269 |
|  | 100 | 3 | 0.10371 | 0.10269 | 0.00190 | 0.00129 | 0.00099 | 0.00028 | 0.00091 | -0.00101 | 0.03009 |

$${}^{a}\bar{e}=(1/N)\sum_{i=1}^{N} e_{i}, \bar{e}_{N}=(1/N)\sum_{i=1}^{N} \bar{e}_{Ri}, \overline{BIAS^{2}}=(1/N)\sum_{i=1}^{N} (\bar{e}_{Ri}-e_{i})^{2}$$
