# Supplementary material for: Estimating misclassification error: a closer look at cross-validation based methods
Source: BMC Res Notes. 2012 Nov 28;5:656. doi: 10.1186/1756-0500-5-656 (PMC3556102; doi:10.1186/1756-0500-5-656)
Supplement: Additional file 4 Table S4 — Simulation results for p = 5, ∑1 = I(5),∑2 = 2I(5),N = 1000. [file 1756-0500-5-656-S4.doc]

Table S4. Simulation results for *p* = 5, $\mathbf{\Sigma}_{1}=I_{(5)},\mathbf{\Sigma}_{2}=2I_{(5)}$, *N* = 1000.

| Method | *n* | $\Delta$ | $\bar{e}$^a^ | $\bar{e}_{N}$ | $\overline{MSE}$ | $SD(MSE)$ | $\overline{VAR}$ | $SD(VAR)$ | $MSB$ | $\overline{BIAS}$ | $SD(BIAS)$ |
| --- | --- | --- | --- | --- | --- | --- | --- | --- | --- | --- | --- |

| LOOCV | 50 | 1 | 0.32393 | 0.32648 | 0.00593 | 0.00832 | 0 | 0 | 0.00593 | 0.00255 | 0.07702 |
| --- | --- | --- | --- | --- | --- | --- | --- | --- | --- | --- | --- |
|  | 50 | 3 | 0.12475 | 0.12530 | 0.00266 | 0.00368 | 0 | 0 | 0.00266 | 0.00055 | 0.05160 |
|  | 100 | 1 | 0.29050 | 0.29190 | 0.00239 | 0.00327 | 0 | 0 | 0.00239 | 0.00140 | 0.04885 |
|  | 100 | 3 | 0.10090 | 0.10126 | 0.00106 | 0.00166 | 0 | 0 | 0.00106 | 0.00036 | 0.03253 |
| BCV*n* | 50 | 1 | 0.32393 | 0.23376 | 0.01512 | 0.00839 | 0.00476 | 0.00150 | 0.01037 | -0.09017 | 0.04731 |
|  | 50 | 3 | 0.12475 | 0.09673 | 0.00403 | 0.00244 | 0.00197 | 0.00090 | 0.00206 | -0.02802 | 0.03574 |
|  | 100 | 1 | 0.29050 | 0.24442 | 0.00583 | 0.00341 | 0.00250 | 0.00065 | 0.00333 | -0.04608 | 0.03478 |
|  | 100 | 3 | 0.10090 | 0.08743 | 0.00178 | 0.00101 | 0.00095 | 0.00034 | 0.00083 | -0.01347 | 0.02550 |
|  |  |  |  |  |  |  |  |  |  |  |  |
| *k*CV*n*/2 | 50 | 1 | 0.32393 | 0.32778 | 0.00602 | 0.00781 | 0.00044 | 0.00020 | 0.00558 | 0.00385 | 0.07461 |
|  | 50 | 3 | 0.12475 | 0.12640 | 0.00274 | 0.00357 | 0.00019 | 0.00012 | 0.00255 | 0.00165 | 0.05051 |
|  | 100 | 1 | 0.29050 | 0.29205 | 0.00237 | 0.00310 | 0.00010 | 0.00005 | 0.00227 | 0.00155 | 0.04763 |
|  | 100 | 3 | 0.10090 | 0.10142 | 0.00106 | 0.00161 | 0.00003 | 0.00002 | 0.00103 | 0.00052 | 0.03205 |
| BCV*n*/2 | 50 | 1 | 0.32393 | 0.23476 | 0.01481 | 0.00821 | 0.00471 | 0.00126 | 0.01010 | -0.08917 | 0.04640 |
|  | 50 | 3 | 0.12475 | 0.09802 | 0.00398 | 0.00239 | 0.00197 | 0.00082 | 0.00201 | -0.02674 | 0.03599 |
|  | 100 | 1 | 0.29050 | 0.24457 | 0.00574 | 0.00331 | 0.00246 | 0.00053 | 0.00328 | -0.04594 | 0.03416 |
|  | 100 | 3 | 0.10090 | 0.08782 | 0.00175 | 0.00100 | 0.00094 | 0.00029 | 0.00081 | -0.01308 | 0.02536 |
|  |  |  |  |  |  |  |  |  |  |  |  |
| *k*CV10 | 50 | 1 | 0.32393 | 0.33285 | 0.00634 | 0.00736 | 0.00109 | 0.00034 | 0.00525 | 0.00892 | 0.07197 |
|  | 50 | 3 | 0.12475 | 0.13081 | 0.00296 | 0.00357 | 0.00048 | 0.00021 | 0.00248 | 0.00605 | 0.04947 |
|  | 100 | 1 | 0.29050 | 0.29565 | 0.00247 | 0.00281 | 0.00040 | 0.00010 | 0.00208 | 0.00514 | 0.04529 |
|  | 100 | 3 | 0.10090 | 0.10356 | 0.00111 | 0.00154 | 0.00013 | 0.00006 | 0.00098 | 0.00266 | 0.03117 |
| BCV10 | 50 | 1 | 0.32393 | 0.23930 | 0.01407 | 0.00772 | 0.00481 | 0.00106 | 0.00927 | -0.08463 | 0.04588 |
|  | 50 | 3 | 0.12475 | 0.10200 | 0.00391 | 0.00225 | 0.00210 | 0.00078 | 0.00181 | -0.02276 | 0.03596 |
|  | 100 | 1 | 0.29050 | 0.24769 | 0.00543 | 0.00315 | 0.00244 | 0.00039 | 0.00299 | -0.04281 | 0.03402 |
|  | 100 | 3 | 0.10090 | 0.08985 | 0.00172 | 0.00097 | 0.00095 | 0.00027 | 0.00076 | -0.01105 | 0.02530 |

$${}^{a}\bar{e}=(1/N)\sum_{i=1}^{N} e_{i}, \bar{e}_{N}=(1/N)\sum_{i=1}^{N} \bar{e}_{Ri}, \overline{BIAS^{2}}=(1/N)\sum_{i=1}^{N} (\bar{e}_{Ri}-e_{i})^{2}$$
